# Insulin resistance, autophagy and apoptosis in patients with polycystic ovary syndrome: Association with PI3K signaling pathway

**DOI:** 10.3389/fendo.2022.1091147

**Published:** 2022-12-16

**Authors:** Cheng Tong, Yue Wu, Lingling Zhang, Ying Yu

**Affiliations:** ^1^ The First Affiliated Hospital of Zhejiang Chinese Medical University (Zhejiang Provincial Hospital of Traditional Chinese Medicine), Hangzhou, Zhejiang, China; ^2^ The First School of Clinical Medicine, Zhejiang Chinese Medical University, Hangzhou, Zhejiang, China

**Keywords:** polycystic ovary syndrome, Insulin Resistance, autophagy, apoptosis, PI3K/Akt/mTOR signaling pathway, ovarian granulosa cells, oxidative Stress

## Abstract

Polycystic ovary syndrome (PCOS) is a disease in which endocrine metabolic abnormalities coexist with reproductive system abnormalities, with the main clinical manifestations including abnormal menstruation, hirsutism, acne, infertility, and obesity, and it is also a high risk for the development of many pregnancy complications, gynecological malignancies and other diseases. Therefore, timely intervention to prevent the progression of PCOS is of great significance for improving the quality of life of most female patients. Insulin resistance (IR) is one of the most common endocrine disorders in PCOS patients, with approximately 75% of PCOS patients experiencing varying degrees of IR. It is now believed that it is mainly related to the PI3K signaling pathway. The role of autophagy and apoptosis of ovarian granulosa cells (GCs) in the pathogenesis of PCOS has also been gradually verified in recent years. Coincidentally, it also seems to be associated with the PI3K signaling pathway. Our aim is to review these relevant studies, to explore the association between the IR, cellular autophagy and apoptosis in PCOS patients and the PI3K pathway. We summarize some of the drug studies that have improved PCOS as well. We have also found that proteomics holds great promise in exploring the pathogenesis of PCOS, and we have published our views on this.

## Introduction

PCOS is one of the most common gynecological disorders in women of reproductive age. It is thought to be a metabolic disease caused by a combination of environmental, endocrine, and genetic causes. Dysbiosis of the intestinal flora has been reported to be associated with the development of PCOS (1). The 2003 Rotterdam Consensus Workshop recognized PCOS as a syndrome of ovarian dysfunction characterized by hyperandrogenemia (HA) and polycystic ovarian changes (2), and the diagnostic criteria require that at least two of the following three characteristics be met: (1) Clinical manifestations or biochemistry suggests hyperandrogenemia, (2) oligo-anovulation, (3) polycystic ovary morphology: ultrasound suggestive of ≥12 follicles with a maximum diameter of 2-9 mm or any ovarian volume >10 ml, meanwhile, other diseases that may cause hyperandrogenemia or reduced ovulation should also be excluded, such as hyperprolactinemia and congenital adrenal hyperplasia, as well as clinical manifestations such as insulin resistance, oligomenorrhea or amenorrhea (3).

## PCOS and complications

In addition, patients with PCOS have a higher incidence of pregnancy complications compared with healthy women, such as miscarriage, gestational hypertension, preterm birth, and infertility (4–12) (**Table 1**). The most serious problems in patients with PCOS of childbearing age are infertility and increased risk of miscarriage by mechanisms such as ovulation disorders, abnormal endocrine metabolism, and impaired egg development (13), however, studies have shown a correlation between these symptoms and the inflammatory response (14). It has been demonstrated that the regulation of PI3K/Akt signaling pathway is associated with inflammation and oxidative stress in granulosa cells of PCOS patients (15). Meanwhile, many studies have found that PCOS may also be closely related to the development of certain common gynecological tumors such as ovarian cancer, breast cancer and endometrial cancer (16). Visfatin mediates malignant transformation of endometrium in PCOS patients through PI3K-Akt and MAPK-ERK signaling pathways (17), which leads to a higher prevalence of endometrial cancer in PCOS patients compared to other women in reproductive age. Also, some patients with PCOS will gradually develop some mental health problems, such as anxiety and depression (18), this may be related to the psychological burden placed on the patient by factors such as the difficulty of curing the disease and infertility.

**Table 1:** Part of reports on the association between PCOS and complications in pregnancy.

Complications	Methods	Results	Reference
**Miscarriage**	A retrospective cohort study with 452 women with recurrent miscarriage and a meta-analysis were conducted.	The prevalence of PCOS in recurrent miscarriage was 9.5%.	(4)
	The study used a nationwide population-based Taiwan National Health Insurance Research Database for the period 1998-2012.	PCOS patients have a 33.5% risk of miscarriage and a 25% risk reduction with metformin treatment.	(5)
	This single-blind randomized controlled trial was conducted at Dr. Shariati Hospital and Omid Fertility Clinic in Tehran, Iran. 178 women with PCOS were included in the study.	The miscarriage rate in the control group (no intervention) was about 33.3%.	(6)
**Gestational hypertension**	The report studied 188 pregnant patients with PCOS treated from June 1, 2018 to November 30, 2020, assessing personal and clinical characteristics of patients with and without gestational hypertension.	The incidence of gestational hypertension in patients with PCOS was 27.66%. BMI ≥ 24 kg/m2, family history of hypertension, history of adverse pregnancy, history of contraceptive use, and family history of gestational hypertension were independent risk factors for the development of gestational hypertension in patients with PCOS, and hypertension is an independent risk factor for the development of gestational hypertension in patients with PCOS.	(7)
	A total of 30 studies eligible for meta-analysis were included.	The association between PCOS and gestational hypertension was significant (OR 2.02, 95CI% 1.83-2.22). This association remained significant after adjusting for age, BMI and infertility (OR 1.48, 95CI% 1.48-1.60).	(8)
	A diverse community cohort study screened 1765 women with PCOS in pregnancy without pre-existing hypertension.	The number of people with hypertension during pregnancy accounted for 48%.	(9)
**Infertility**	This is a large community-based cohort study with 9145 respondents aged 28-33 years.	72% of women with polycystic ovary syndrome had infertility, while only 16% of women without polycystic ovary syndrome were infertile.	(10)
**Preterm birth**	This study included 1,167 women diagnosed with PCOS using the Rotterdam criteria, underwent a frozen embryo transfer (FET).	Women with polycystic ovary syndrome have a higher chance of preterm delivery compared to women without polycystic ovary syndrome(OR1.53, 95% CI1.23-1.91).	(11)
	This study performed a clinical examination, including transvaginal ultrasound, on pregnant women who met the inclusion criteria.	Twenty-nine of 114 women (25.4%) with preterm delivery met the criteria for PCOS, while 18 of 127 women (14.2%) with full-term pregnancy met the criteria for PCOS (P = 0.03).	(12)

## Insulin resistance

IR is a metabolic disorder in which an individual’s sensitivity to exogenous or endogenous insulin is reduced, resulting in the body’s inability to use insulin to adjust blood glucose levels, leaving blood glucose at a consistently high level. IR is a complex metabolic disorder that is a key risk factor for many diseases such as cardiovascular disease, and can lead to obesity and type 2 diabetes (19), etc. Large-scale genome association studies have been conducted in recent years to help us identify common genetic variants associated with IR (20). It is now generally accepted that there is an association between the presence of reduced insulin sensitivity and pancreatic islet beta cells (21).

## Autophagy and apoptosis

In the past, it was widely believed that cell death was mainly in the form of apoptosis and necrosis, however, recent studies have found that autophagy is also a pathway of cell death. Autophagy affects cell survival by maintaining cellular bioenergy and removing proteins and damaged organelles (22). A study reveals the important role of cellular autophagy in regulating cellular metabolism after knocking out autophagy-related genes in mice (23). In the ovary, oocytes cannot develop without autophagy (24), and when follicle cell autophagy is defective, it can lead to female infertility (25). Autophagy plays a significant role in the role of the insulin signaling pathway, and its role differs for different target organs of the INS pathway. In skeletal muscle and liver, autophagy stimulates INS pathway activity, while in the pancreas, autophagy destroys pancreatic β-cells to induce insulin resistance, thus suggesting that autophagy can regulate the role of INS (26). Apoptosis is characterized by a number of characteristic morphological changes in cell structure that result in the clearance of cells from the body and the absence of spillage of cell contents into the environment with minimal damage to surrounding tissues (27). Studies in recent years have concluded that some pathophysiological changes in PCOS are inseparable from autophagy and apoptosis of ovarian granulosa cells (GCs).

## PI3K/AKT/mTOR signaling pathway

**Figure 1:**
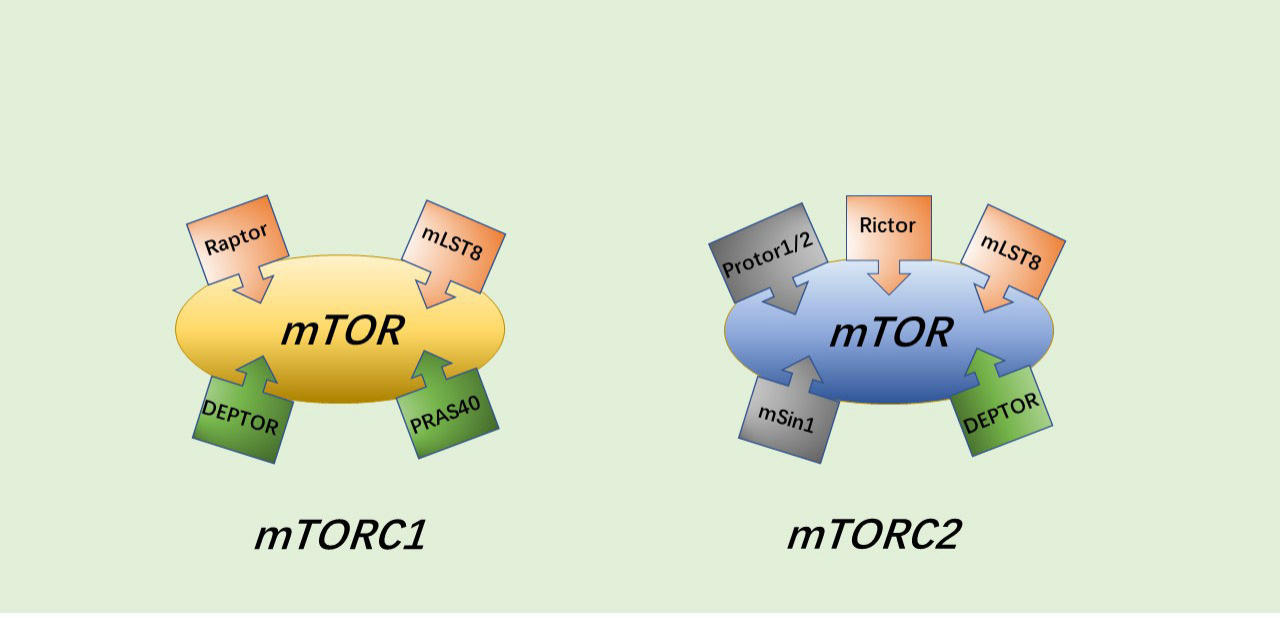
The general structure of mTORC1 and mTORC2.

Current research has revealed that the PI3K/AKT/mTOR pathway has an important biological role in protein and glycogen synthesis and in regulating cell activity, senescence and death (28–29). In addition to endocrine metabolic abnormalities (30), disorders of this pathway are also associated with various malignancies, leukemia, schizophrenia, coronary heart disease, heart failure, osteoarthritis and other diseases (31–36). The PI3K/AKT/mTOR pathway can be activated by multiple types of stimuli to play a role in inhibiting apoptosis and autophagy, and promoting cell growth, with AKT, a serine/threonine kinase, being the core of the entire PI3K/AKT/mTOR pathway. mTORC1 and mTORC2 are two different protein complexes formed by the target of rapamycin (mTOR). mTORC1 has three core components: mTOR, Raptor and mLST8. However, in addition to the three core components, PRAS40 and DEPTOR play some roles as two inhibitory subunits as well. The composition of mTORC2 is similar to that of mTORC1, containing DEPTOR, mSin1 and Protor1/2 in addition to mTOR, mLST8 and Rictor (which may have a similar function to Raptor) (37) (**Figure 1**).

**Figure 2:**
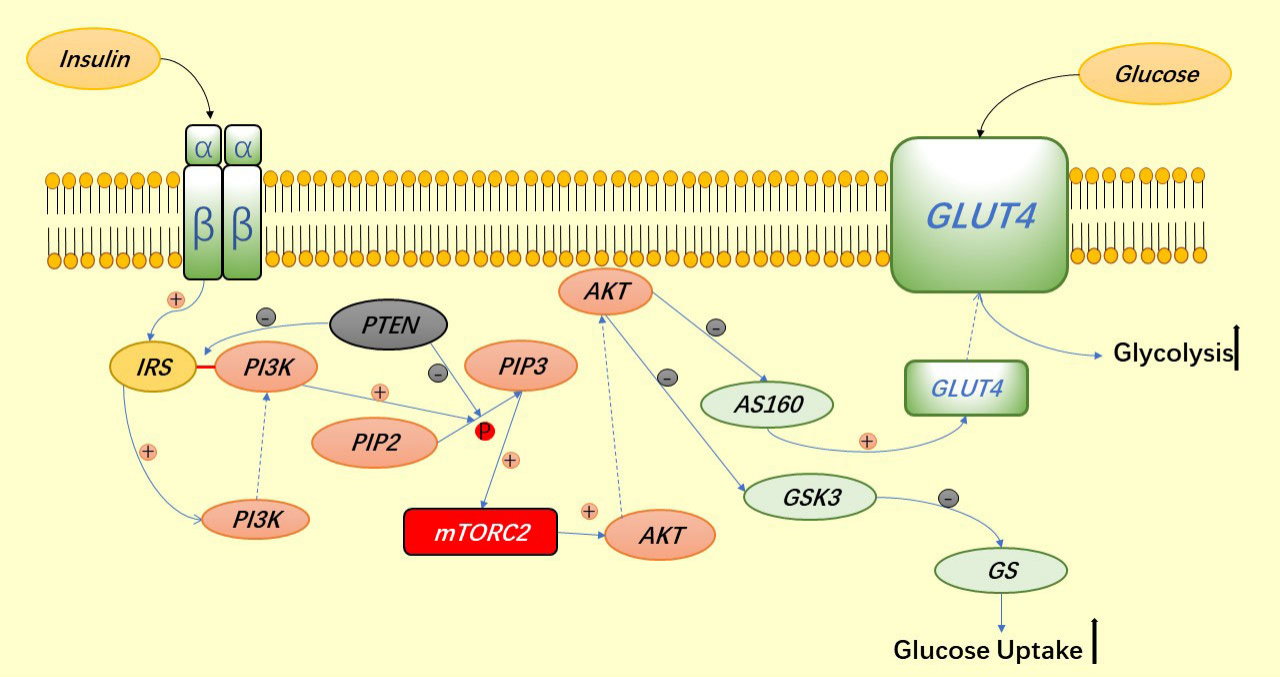
The PI3K signaling pathway involved in glucose uptake.

The insulin receptor is a tetrameric structure consisting of 2 α subunits and 2 β subunits. When insulin binds to the α subunits, the β subunits become activated, auto phosphorylate themselves and activate the insulin receptor substrate (IRS) (38), however, this process is regulated by PTEN dephosphorylation. IRS activation leads to P13K recruitment and binding to the IRS, after which P13K phosphorylates PIP2 to become PIP3, but similarly, PTEN can dephosphorylate to regulate this process (39). Increased concentrations of PIP3 are followed by recruitment of AKT in response to mTORC2, and AKT activation not only phosphorylates and inhibits AS160 (an inhibitor of GLUT4 transport), which means that when AKT inhibits AS160, it facilitates GLUT4 aggregation on the cell membrane and thus promotes glucose entry into the cell for glycolysis (37), GS is a glycogen synthase and GSK3 is a natural inhibitor of GS, which inhibits GS phosphorylation and prevents it from functioning, but AKT can directly inhibit GSK3 and thus promote glycogen synthesis (40) (**Figure 2**).

**Figure 3:**
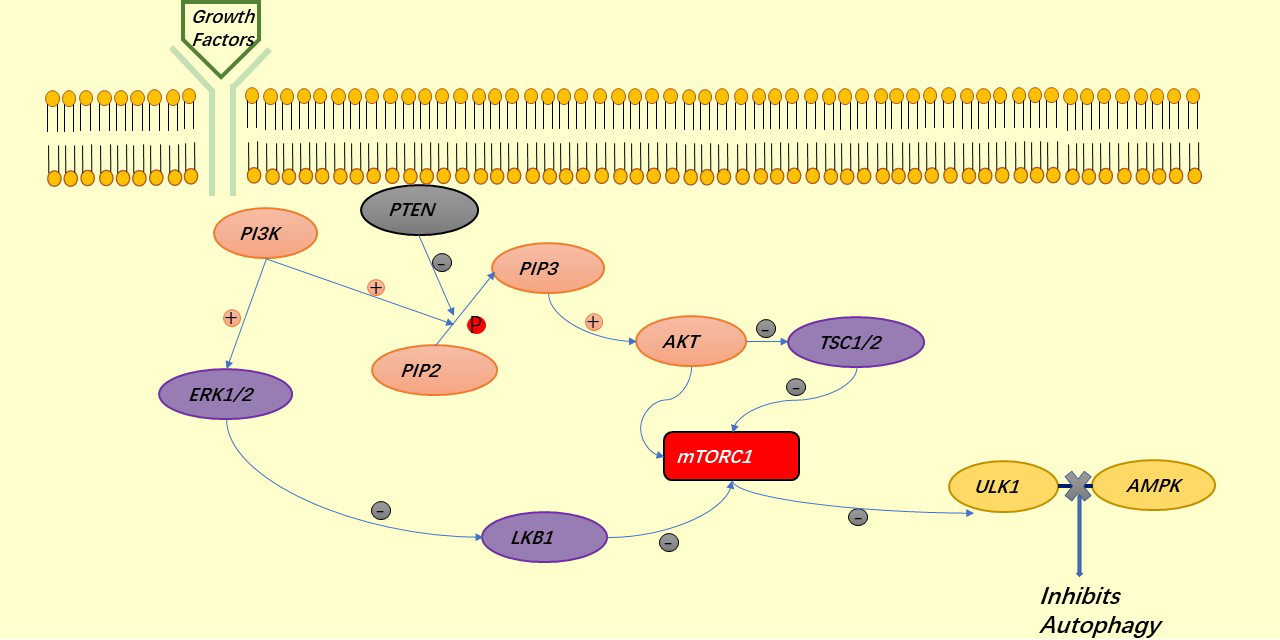
The PI3K signaling pathway involved in the regulation of autophagy.

**Figure 4:**
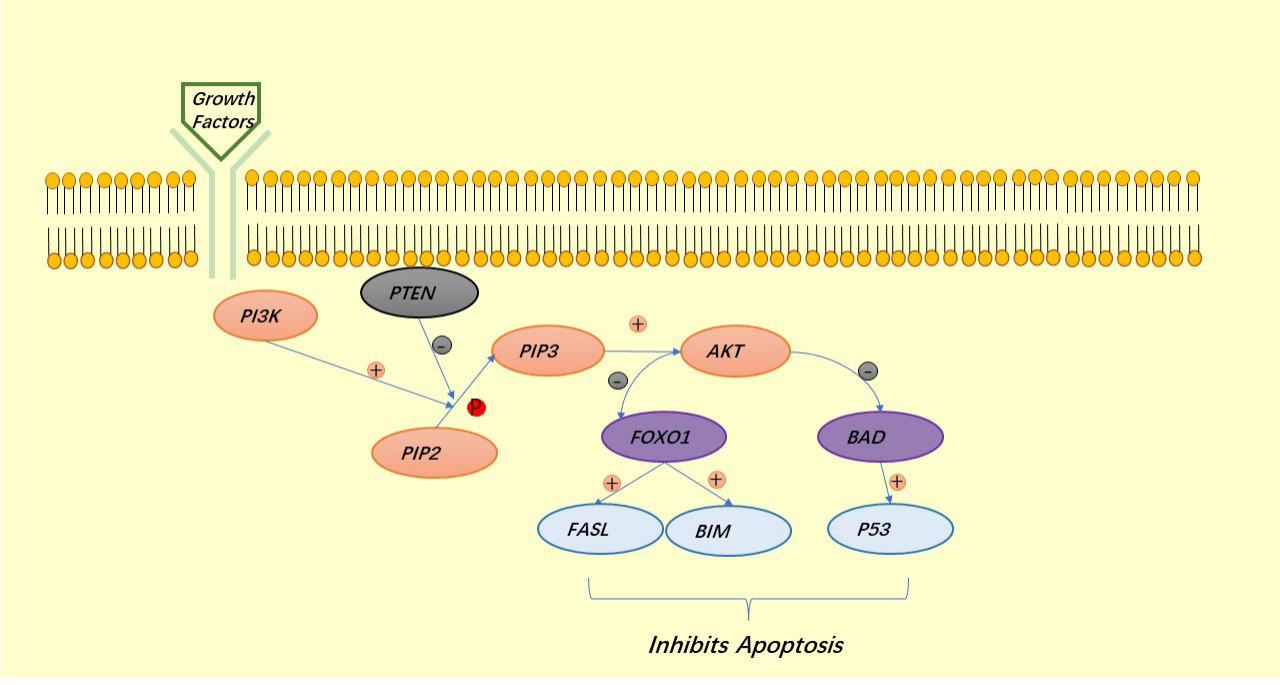
The PI3K signaling pathway involved in the regulation of apoptosis.

AKT is a major regulator of cell survival. TSC1/2 is an upstream inhibitor of mTORC1, which is also a negative regulator of autophagy and is regulated by AKT, thus exerting a role in the inhibition of autophagy by mTORC1:mTORC1 blocks the binding of ULK1 to AMPK (34). Under the stimulation of growth factors, LKB1 is regulated by the upstream signaling factors PI3K and ERK1/2, thus deregulating mTORC1 and also exerting the role of mTORC1 in inhibiting autophagy (41). AKT can also directly regulate mTORC1 to prevent autophagy (42, 43) (**Figure 3**). Similar to autophagy, apoptosis is regulated by AKT. AKT inhibits apoptosis either directly by inhibiting pro-apoptotic proteins (BAD) or by inhibiting pro-apoptotic signals generated by FOXO1, of which the downstream signals are p53 for the former and FASL and BIM for the latter (40) (**Figure 4**).

## IR in PCOS

IR is prevalent in patients with PCOS, and to date, the internationally accepted method for measuring insulin sensitivity is the hyperinsulin orthoglycemic clamp. According to statistics, about 75% of PCOS patients develop IR, with a higher percentage of PCOS patients with obesity, and the development of PCOS is also inseparable from IR (3). It is now believed that some clinical manifestations such as hyperandrogenemia and obesity occur in PCOS patients are closely related to IR, and alarmingly, insulin resistance and hyperandrogenism interact with each other to worsen the disease. There is evidence that IR exacerbates hyperandrogenemia, causes disturbances in the regulation of the hypothalamic-pituitary-ovarian axis, and accelerates the progression of PCOS (44). However, large amounts of androgens in the body can also affect the action of insulin, thus reducing the body’s sensitivity to insulin action and leading to IR, and can even affect the function of pancreatic beta cells, leading to a decrease in the body’s sensitivity to insulin (45). Excess androgens in the human circulation lead to fat deposition in various organs, causing IR and hyperinsulinemia, which promotes androgen secretion from the ovaries and adrenal glands. This cyclic interaction between IR and HA can further lead to ovarian dysfunction, causing anovulation and infertility. Therefore, it can be inferred that it is the interaction of high levels of androgens and high levels of insulin that together lead to a series of physiological disorders in PCOS patients (46), and IR is also a bridge to various metabolic disorders in PCOS patients, so early intervention or prevention of IR in PCOS patients who present with IR can be beneficial to patients.

## The PI3K signaling pathway and IR in PCOS

IR is one of the important features of PCOS, and although it is only part of the pathogenesis of PCOS, many scholars regard it as a key factor in the pathogenesis of PCOS and is likely to be related to the PI3K signaling pathway. The PI3K pathway is not only involved in regulating cell growth, proliferation, and metabolism (47), but is also implicated in the development of some tumors, such as non-small cell lung cancer (48), bladder cancer (49), and osteosarcoma (50). Insulin activates the PI3K signaling pathway and auto phosphorylates the receptor to PI-3,4-diphosphate, or PI-3,4,5-triphosphate, and when the insulin receptor is activated, its binding to the IRS promotes its binding to PI3K, thus exerting its effect (51). Investigators established an animal model of insulin resistance by injecting neuropeptide Y into the paraventricular nucleus of the hypothalamus of rats, and performed glucose tolerance tests after 8 weeks, while evaluating the adipose tissue of rats. The results revealed reduced expression of pGSK3a and pGSK3b in their PI3K/Akt signaling pathway, suggesting that IR is related to the PI3K/Akt signaling path (52). Abuelezz (53) et al. reported for the first time the role of PI3K/AKT/mTOR in the pancreas of patients with PCOS: low expression of the PI3K/AKT/mTOR pathway in the pancreas leads to impaired insulin sensitivity, decreased β-cell function and quality, and thus IR, and also demonstrated for the first time that nanocurcumin restores insulin sensitivity in patients with PCOS effective effect. The PI3K signaling pathway is not only important for the development of IR in patients with PCOS, but is likewise closely associated with the progression of PCOS. It has been demonstrated that the P13K/AKT pathway underlies the development of endometrial malignant lesions in patients with PCOS combined with IR (54). This is yet another reminder of the importance of timely intervention in IR in patients with PCOS to improve their prognosis.

In recent years, studies related to the mechanism of action of PI3K signaling pathway to improve IR in PCOS patients have been gradually reported. Melatonin significantly upregulated the expression of insulin receptor substrate 1 (IRS-1) and glucose transporter protein 4 (GLUT4), improved glucose uptake in PCOS patients, increased p-PI3K and p-Akt levels, and reduced IR in human ovarian granulosa cells (GCs) and palmitic acid (PA)-induced cells through the PI3K/Akt signaling pathway (55). Sharma (56)et al. found that Caulerpa lentillifera extract (CLE), an alga used for medicinal purposes, can regulate glucose uptake in mice through the PI3K/AKT signaling pathway. CLE significantly reduced HOMA-IR, fasting glucose, and plasma insulin levels in model mice and significantly enhanced IRS, AKT, PI3K, and GLUT4 activation, however, the ability of CLE to increase glucose uptake was abolished after treatment with a PI3K inhibitor (LY294002). Gallic acid has been shown to have a similar effect of enhancing glucose uptake *via* the PI3K/Akt signaling pathway in animal models (57). Berberine is a compound prepared by extracting the active ingredients from various plants and has been used in traditional Chinese medicine for many years to treat endocrine disorders and infertility (58). The reduction of HOMA-IR index by berberine was confirmed in PCOS rats through activation of PI3K/Akt and inhibition of MAPK pathway (59). Liu Wei Di Huang Wan (A tablet made from six traditional Chinese medicines) can reduce insulin resistance in PCOS rats by improving dual regulation of PI3K/AKT and MAPK pathways (60). The hypoglycemic effect of tea polysaccharide is achieved by enhancing the expression of PI3Kp85-p-Akt-GLUT4 (61). Further studies on metformin revealed that its mechanism of action, in addition to being an adenosine monophosphate-activated protein kinase activator (62), can benefit PCOS patients by improving the PI3K pathway (63). These experiments not only confirmed the clear role of PI3K signaling pathway in IR, but also provided more evidence for clinical treatments of IR in PCOS patients.

## Autophagy and apoptosis in PCOS are associated with PI3K signaling pathway

mTOR is a serine/threonine protein kinase that controls cell division, autophagy, maturation and apoptosis through the PI3K/AKT/mTOR pathway and is also involved in tumor metastasis. Autophagy is the process by which protein components in cells are degraded by lysosomes, thereby maintaining new protein and energy production to keep the body alive (64), and this process can be initiated by a variety of stressors and is a mechanism for killing stress cells and maintaining homeostasis in the body’s internal environment (27). The production of apoptosis is associated with stimulation of the cell leading to changes in the mitochondrial membrane, allowing pro-apoptotic proteins to pass through the mitochondria into the cytoplasm (65). Both apoptosis and autophagy are important ways to ensure the orderly renewal of cells and maintain the stability of the internal environment of the organism. Studies in recent years have found that some pathological changes in PCOS seem to be related to the disruption of this balance, in which the PI3K pathway plays a significant role. Recently, clinical studies have demonstrated significant autophagy in ovarian granulosa cells (GCS) of PCOS patients (66). Choi (67)et al. found that after inhibition of the PI3K/AKT/MTOR pathway, autophagy was activated leading to apoptosis and degeneration of GCs, which in turn triggered follicular atresia. Oxidative stress is one of the main causes of autophagy and apoptosis in cells. Gong (68)et al. demonstrated that oxidative stress mechanisms induce apoptosis and downregulate the PI3K/Akt signaling pathway in PCOS patients, but growth hormone can antagonize this effect and restore PI3K/AKT pathway activity. Shen (69)et al. found that follicle-stimulating hormone (FSH) can inhibit autophagy in GCs caused by oxidative stress, however, it is predicated on the activation of the PI3K-AKT-mTOR signaling pathway and, one study found that blocking this pathway after administration of mTOR blockers induced autophagy (70). Dehydroepiandrosterone (DHEA)-induced PCOS mice exhibit circulating blood and skeletal muscle IR, along with activation of mTORC1 and inhibition of autophagy in mouse skeletal muscle. Dysregulation of the mTOR pathway causes autophagy leading to mitochondrial damage and reduced glucose uptake, ultimately leading to hyper-androgen-induced skeletal muscle IR (71). This experiment may explain why PCOS patients often have a combination of both hyperandrogenemia and insulin resistance. It is clear from the above experiments that the PI3K signaling pathway is essential for the regulation of both autophagy and apoptosis, and that this mechanism is also relevant in patients with PCOS and contributes to a range of other complications such as IR. However, further research is needed to understand the cause of the abnormal activity of this pathway in PCOS patients and which part of the pathway is dysregulated, leading to a range of pathophysiological changes in PCOS patients, which may be an important part of the pathogenesis of PCOS. Some drugs have been reported to modulate autophagy and apoptosis in PCOS *via* the PI3K pathway, thereby alleviating some of the pathological changes in PCOS. In model rats treated with Bu-shen-zhu-yun decoction (a traditional Chinese herbal remedy for infertility), protein expression in the PI3K/AKT/mTOR pathway was significantly upregulated, while apoptosis-related proteins were significantly downregulated in PCOS rats, suggesting that apoptosis can be improved by enhancing PI3K/AKT/mTOR protein expression (72). Xie (73) et al. showed that melatonin improved ovarian dysfunction by regulating autophagy in DHEA-induced PCOS through the PI3K-Akt pathway. Gui Zhi Fu Ling Wan is also a traditional Chinese medicine used to promote blood flow and prevent blood stagnation for the treatment of many gynecological conditions. This drug can inhibit GCs cell autophagy and promote follicle development to alleviate ovulation disorders in PCOS combined with IR rats, which is associated with the activation of PI3K/AKT/mTOR signaling pathway by the drug (74). The role of metformin in improving hyperglycemia, restoring insulin sensitivity and improving hyperandrogenemia in patients with PCOS is now well established (75), however, it has also been found to improve ovarian function in patients, possibly through a P13K/AKT/mTOR pathway that resists cellular autophagy (76). Further studies suggest that this mechanism is achieved through the P13K/AKT/mTOR signaling pathway that reduces the level of oxidative stress in GCs and upregulates antioxidant enzymes (77). But non-pharmacological means of treatment have been reported to have the opposite result. After acupuncture treatment, the ovarian morphology of 90% of the rats in the experimental group was significantly improved, and the expression of PI3K, AKT, mTOR, and LncMEG3 in the ovarian granulosa cells of the rats was found to be decreased after acupuncture, while the expression of follicle stimulating hormone receptor was increased (78). This result suggests that acupuncture can inhibit the PI3K/AKT/mTOR pathway, reduce granulosa cell autophagy, and normalize their proliferation. While the aforementioned drug studies confirm that the main mechanism by which they inhibit autophagy and apoptosis is by improving PI3K signaling pathway activity, non-drug research protocols (like acupuncture) report the opposite experimental conclusion: that by inhibiting the PI3K signaling pathway, they inhibit autophagy and apoptosis. This is an interesting phenomenon and also warrants further research by scholars to elucidate the reasons for the two opposing findings.

## The use and future of proteomics in PCOS

Qualitative and quantitative analysis of proteins, whose biological properties dictate their involvement in different physiological processes, can provide an advantage for research in various fields worldwide. Proteomics is an emerging technique based on liquid chromatography-tandem mass spectrometry (LC-MS/MS) that provides direct information about proteins rather than RNA, and therefore offers advantages over transcriptomics (79). This technology is already playing an important role in basic research in several disciplines (80). In our review of the literature, we found that proteomics is gradually being used in the study of PCOS. Recently, 80 proteins that may be associated with PCOS were screened and analyzed in the sera of PCOS patients. 56 down-regulated proteins and 24 up-regulated proteins were identified, and PRDX1 was one of the up-regulated proteins with a large area under the ROC curve, suggesting that it has a good diagnostic value for PCOS (81). In an *in vitro* study of HEPG2 cells induced by sodium palmitate to develop insulin resistance, experiments found that insulin sensitivity was significantly enhanced after knockdown of PRDX1 expression by siRNA, while insulin sensitivity of the cells was significantly reduced when PRDX1 was overexpressed, and they found that P38MAPK expression was significantly enhanced in cells induced under these conditions, so it can be tentatively concluded that PRDX1 can induce IR (82). However, whether this conclusion holds true in PCOS and whether PRDX1 affects the PI3K signaling pathway are questions that need to be further investigated. Overall, whether proteomics techniques can be used to screen for proteins with diagnostic or even therapeutic value in PCOS, to confirm their pathogenesis in PCOS through further studies, and to explore effective treatments (like corresponding targeted drug therapy) based on these mechanisms may be an important part of future research and prevention of PCOS.

## Conclusion and future

PCOS is a gynecological condition that afflicts many women of childbearing age and has a variety of complications, but the causes are still not fully understood and there are no clear clinical criteria for the diagnosis of PCOS, which is still mostly implemented with reference to the Rotterdam Diagnostic Criteria. PCOS is mainly characterized by endocrine disruption and ovulation disorders. Although many scholars at home and abroad have been investigating its pathogenesis, PCOS is a complex metabolic syndrome with a variety of clinical manifestations that may involve multiple mechanisms, so an in-depth study of the pathogenesis of PCOS is of great significance for the diagnosis and treatment of PCOS. The PI3K signaling pathway is one of the most intensively studied signaling pathways and is associated not only with the development of IR in PCOS, but also with autophagy and apoptosis. Although there are experimental findings to the contrary, there is no denying the important role that the PI3K pathway plays in this. How to prevent the development of PCOS and reduce the impact of PCOS on patients remains a challenge that needs to be addressed. We have mentioned above a number of studies that have improved insulin resistance and autophagy in PCOS through drugs, mainly by directly improving the activity of the PI3K pathway or by increasing the antioxidant capacity of the cells. But which one or several signaling proteins of the pathway these drugs act on, and what the specific mechanisms by which it acts on the pathway are, need further study. We then raised the prospect of applying proteomics to the study of PCOS, which has many advantages and can be used for qualitative or quantitative studies of proteins. We then raised the prospect of applying proteomics to the study of PCOS, a discipline that explores the full range of protein species and modifications in the organism under specific conditions, and which has many advantages for exploring the specific properties of proteins. We therefore believe that the integration of proteomics with current research may be a direction for future PCOS research and may also inform research into the prevention and treatment of other chronic diseases.

## Author contributions

CT and YW came up with the idea. CT wrote the initial draft (including substantive translation), made the table and the figure. YW contributed to the preparation of the published work. LZ contributed to language revision. YY ensured that the descriptions are accurate and agreed by all authors. All authors contributed to the article and approved the submitted version.
